# Ablative fractional laser treatment reduces hedgehog pathway gene expression in murine basal cell carcinomas

**DOI:** 10.1007/s10103-024-03997-1

**Published:** 2024-02-03

**Authors:** Kristian Kåber Pedersen, Jonatan Riber Granborg, Catharina Margrethe Lerche, Thomas Litman, Uffe Høgh Olesen, Merete Hædersdal

**Affiliations:** 1 Department of Dermatology, Copenhagen University Hospital Bispebjerg, 2400 Copenhagen, Denmark; 2 Department of Pharmacy, University of Copenhagen, 2100 Copenhagen, Denmark; 3 Department of Immunology and Microbiology, University of Copenhagen, 2200 Copenhagen, Denmark; 4 Molecular Biomedicine, LEO Pharma A/S, 2750 Ballerup, Denmark

**Keywords:** Ablative fractional laser, Basal cell carcinoma, Hedgehog inhibitors, Vismodegib, Murine tumor model

## Abstract

This study aimed to investigate the impact of ablative fractional laser (AFL) on hedgehog pathway gene expression in murine microscopic basal cell carcinomas (BCCs) and compare these results to the effect of topical treatment with vismodegib, an FDA-approved hedgehog inhibitor. In 25 mice, 1 cm^2^ skin test sites (*n* = 44) containing microscopic BCCs were exposed to one of three interventions: a single CO_2_ AFL treatment (1 pulse, 40 mJ/microbeam, wavelength 10.6 μm, 5% density, pulse rate 250 Hz, *n* = 12), eight topical vismodegib treatments (3.8 mg/mL, *n* = 8), or combination of AFL and vismodegib treatments (*n* = 9). Untreated controls were included for comparison (*n* = 15). After 4 days, skin samples were analyzed for hedgehog gene expression (*Gli1*, *Gli2*, and *Ptch1*) by qPCR and vismodegib concentrations by liquid chromatography mass spectrometry (data analyzed with two-tailed *t*-tests and linear regression). A single treatment with AFL monotherapy significantly reduced hedgehog gene expression compared to untreated controls (*Gli1* 72.4% reduction, *p* = 0.003; *Gli2* 55.2%, *p* = 0.010; *Ptch1* 70.9%, *p* < 0.001). Vismodegib treatment also reduced hedgehog gene expression (*Gli1* 91.6%; *Gli2* 83.3%; *Ptch1* 83.0%), significantly surpassing AFL monotherapy for two out of three genes (*Gli1*, *p* = 0.017; *Gli2*, *p* = 0.007; *Ptch1*, *p* = 0.15). AFL and vismodegib combination mirrored the effects of vismodegib monotherapy (*Gli1*, *p* = 0.424; *Gli2*, *p* = 0.289; *Ptch1*, *p* = 0.593), possibly due to comparable cutaneous vismodegib concentrations (mean ± SD, vismodegib monotherapy 850 ± 475 µmol/L; combination 1036 ± 824 µmol/L; *p* = 0.573). In conclusion, a single AFL treatment significantly reduced hedgehog gene expression in murine BCCs mimicking the effects of eight topical applications of vismodegib. Further studies are needed to assess whether AFL can be utilized for BCC treatment, either as monotherapy or in combination with other drugs.

**Supplementary information:**

The online version contains supplementary material available at 10.1007/s10103-024-03997-1.

## Introduction

Basal cell carcinoma (BCC) is the most common keratinocyte skin cancer with five million new cases in the US each year [1, 2]. Most basal cell carcinomas are treated surgically, which may lead to disfiguring cosmetic appearances, in particular for patients with multiple lesions. Non-surgical treatments hold potential to improve cosmetic outcomes from skin cancer treatment [3]. The hedgehog inhibitors, vismodegib and sonidegib, have received approval by the US Food and Drug Administration for treatment of advanced and metastatic BCCs, and can be used as alternatives to surgical treatments [4]. Vismodegib and sonidegib specifically inhibit the hedgehog signaling pathway, which is essential for BCCs, but their clinical use is limited by adverse effects caused by systemic hedgehog inhibition [4–6]. Topical treatment may reduce side effects and improve patient tolerability. Preclinical studies have shown that both chemical and physical penetration enhancers such as emulsion formulation of vismodegib and ablative fractional laser (AFL) increase cutaneous hedgehog inhibitor uptake, but few studies have investigated the effect of increased cutaneous vismodegib levels on hedgehog pathway genes, and no studies have looked into the effects of AFL treatment alone [7].

Previous studies have shown that AFL treatment leads to many different tissue responses ranging from wound healing activation [8, 9] and immune cell recruitment [10, 11] to skin cancer prevention [12–14]. On a cellular level, these responses are associated with changes in expression levels of various genes [8, 9, 14]. We hypothesized that AFL treatment could result in hedgehog pathway inhibition. Therefore, this study aimed to investigate the impact of AFL treatment on hedgehog pathway gene expression in microscopic murine BCCs and to compare these results to the effect of topical vismodegib treatment.

## Methods

### Animals

The study was approved by the Danish Animal Experiments Inspectorate (protocol code 2019-15-0201-01666 of 12 May 2019). Transgenic female mice with the genotype: Ptch1^+/-^ K14-CreER2 p53^fl/fl^ [15] were used for this study (*n* = 30). At age 20–31 weeks, we induced BCC tumors in some of the mice (*n* = 25) by applying a single dorsal full-body X-ray irradiation of 4 Gy at 50 kV over a period of 2.05 min (Model D3100, Gulmay Medical, Surrey, Britain) followed by three intraperitoneal injections of 300 µg tamoxifen over 3 days (Sigma-Aldrich, SKU T2859-1G, Munich, Germany). After 4–6 months, visible BCCs appeared, hedgehog genes were upregulated, and microtumors showed up in histological samples (Figure 2A). Mice were anesthetized with a combination of midazolam (2.5 mg/mL, Hameln®, Region Hovedstadens Apotek, Copenhagen, Denmark) and a mix of fentanyl citrate (0.158 mg/mL) and fluanisone (5 mg/mL, Hypnorm® Vet, Skanderborg Apotek, Skanderborg, Denmark) during x-ray and AFL procedures. Following treatment, each mouse was checked daily until termination.

### Study design

**Fig. 1:**
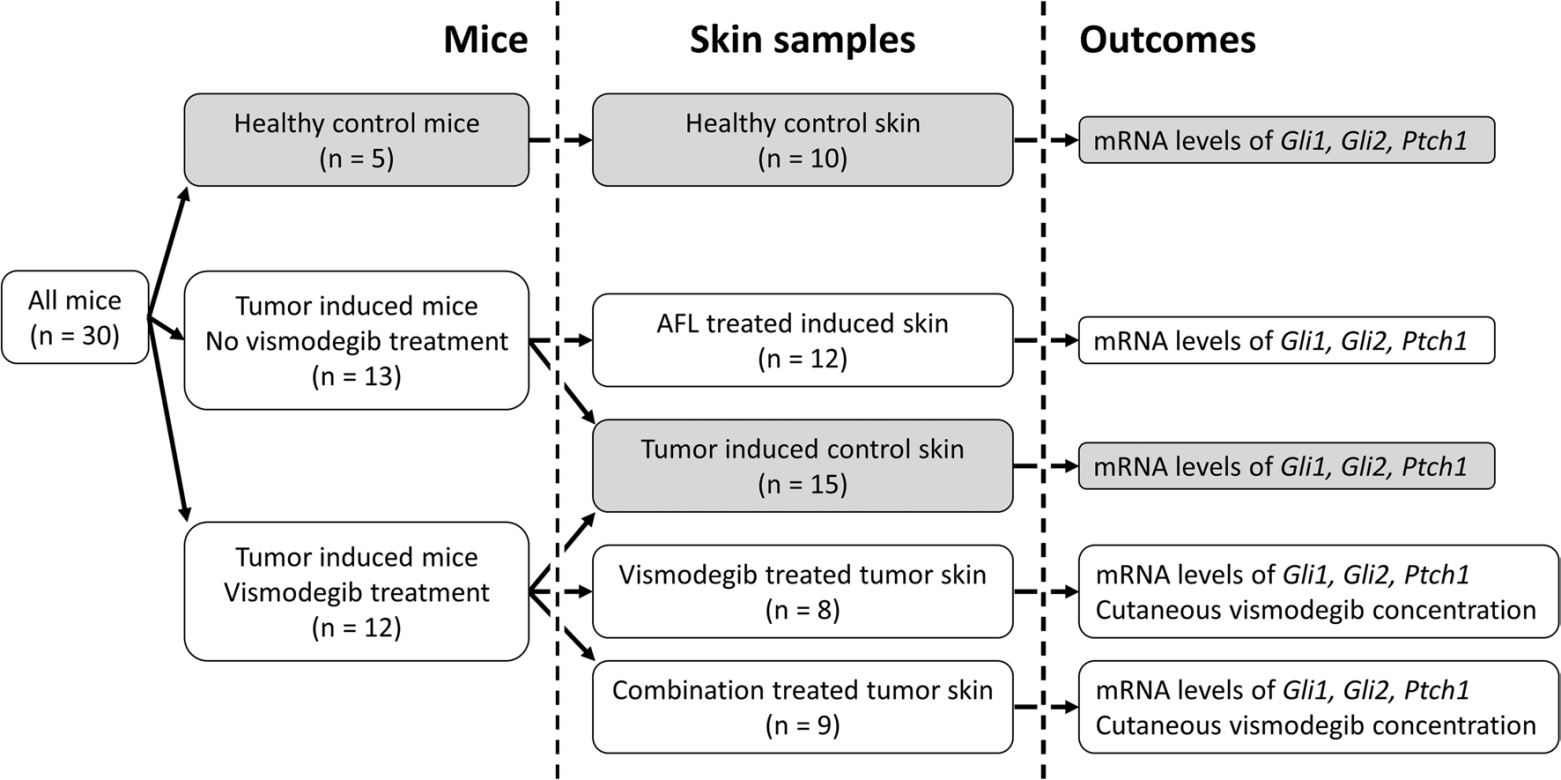
Overview of the study. A total of 54 samples were generated from 30 mice. Grey boxes indicate control groups

An overview of the study can be seen in Figure 1. Tumor-induced murine skin with microscopic BCCs was exposed to one of three treatments: (i) a single treatment with AFL monotherapy (*n* = 12); (ii) eight topical applications of vismodegib emulsion (*n* = 8); (iii) a combination of the AFL and vismodegib treatments (*n* = 9). Controls included tumor-induced murine skin (*n* = 15) and healthy skin (*n* = 10). The outcome measures included qPCR analysis of mRNA expression of the hedgehog genes *Gli1*, *Gli2*, and *Ptch1*; liquid chromatography tandem mass spectrometry (LC-MS/MS) quantification of skin vismodegib concentrations; and histological analysis of AFL channels and microscopic tumors.

### AFL treatment and vismodegib emulsion application

Mice receiving AFL treatment were exposed to a single treatment with a 10,600 nm fractional CO_2_-laser (Ultrapulse, DeepFx handpiece, Lumenis, Santa Clara, CA, USA) set to 40 mJ/microbeam and 5% density on day 0 (1 pulse, wavelength 10.6 μm, pulse rate 250 Hz). Vismodegib treatment was initiated 15–30 min after AFL treatment and consisted of a total of eight treatments, two treatments per day, with 15 µL of topical vismodegib (Erivedge®, Genentech, San Francisco, CA, USA) in a sustained release microemulsion previously developed in-house [16, 17]. To ensure that vismodegib did not diffuse to other test sites, interventions that included vismodegib were performed on separate mice.

### Biopsy isolation

On day 4, 4 h after the last vismodegib application, the experiment was terminated. From 12 vismodegib-treated mice and three control mice, 150–950 µl intra-cardiac blood was sampled and centrifuged 2000 *g* (10 min, 4 °C; SIGMA, Osterode am Harz, Germany) to isolate plasma for measurement of systemic vismodegib concentrations. 1 cm^2^ skin test sites were tape stripped thrice (3M Tegaderm™, Copenhagen, Denmark) to remove superficial vismodegib before being cut into 2 halves with a scalpel (0.5 cm^2^ pieces, Kiato, Mediq Danmark, Denmark). One-half was used for qPCR analysis and the other for vismodegib concentration measurement. Samples for qPCR analysis were submerged in 1 mL RNAlater (Thermo Fisher/Invitrogen, catalog number AM7021, Vilnius, Lithuania) and stored at 4 °C, and samples for vismodegib measurements were weighed (AB204, Mettler Toledo, Im Langacher, Switzerland) and stored at − 80 °C.

### qPCR analysis

Skin samples were removed from RNAlater solution, cut into small pieces, transferred to TRIzol reagent (Thermo Fisher/Invitrogen, catalog number 15596018, Carlsbad, CA, USA) and homogenized in a Retsch MM400 mixer mill (RRID:SCR_020427, Retsch, Haan, Germany) for 30 min. RNA was released from the TRIzol solution using Acid-Phenol:Chloroform (Thermo Fisher/Invitrogen, catalog number AM9722, Carlsbad, CA, USA) and purified with the Nucleospin RNA kit, which includes DNAse treatment (Macherey-Nagel, item number 740933.250, Düren, Germany). Then, RNA concentration was measured with a Nanodrop 1000 (RRID:SCR_016517, Thermo Scientific, Waltham, MA, USA) and first-strand cDNA was synthetized using the iScript cDNA synthesis kit (BioRad, product number 1708891, Hercules, CA, USA) from 900 ng of RNA per sample. qPCR was performed in duplicate in a Stratagene MX3005P thermocycler (RRID:SCR_019526, Agilent Technologies, Waldbronn, Germany) using TaqMan Universal PCR Master Mix No Amperase (Thermo Fisher/Applied Biosystems, Warrington, UK) and TAQman probes (Thermo Fisher/Applied Biosystems, Warrington, UK; Probe IDs are listed in Supplementary Information in Supplementary Table 1). *Gli1*, *Gli2*, and *Ptch1* mRNA levels were normalized to the geometric mean of three housekeeping genes: *Rplp0*, *Rpl19*, and *Ppia*. Results are reported as gene expression in a single skin test site compared to the geometric mean of gene expression in skin from healthy control mice.

### LC-MS/MS vismodegib quantification

Excised skin was mixed with 900 µL DMSO (SIGMA, Steinheim, Germany) + 100 µL DMSO:H_2_O (1:1 v/v). Calibration curves were prepared by adding known amounts of vismodegib to the 100 µL DMSO:H_2_O (1:1 v/v). After mixing, the samples were homogenized with grinding balls at 30 Hz for 1 h 30 min at 4 °C using the Retsch MM400 mixer mill. Tissue homogenates were vortexed and centrifuged at 10,000 *g* for 10 min. Then, 100 µL of the supernatants was mixed with 100 µL H_2_O and centrifuged again. One hundred microliters of the new supernatants was diluted by a factor of 500 in DMSO:H_2_O (1:1 v/v) and analyzed by LC-MS/MS. The lower limit of quantification of vismodegib in skin was 3 ng/mL.

Blood plasma samples were treated with acetonitrile for protein precipitation proteins: 80 µL of blood plasma was mixed with 20 µL DMSO:H_2_O and 80 µL acetonitrile, and centrifuged at 15,000 *g* for 10 min at 4 °C. Calibration curves were prepared by adding known amounts of vismodegib in 20 µL DMSO:H2O (1:1 v/v). One hundred microliters of the supernatants was diluted by a factor of 10 and analyzed by LC-MS/MS. The lower limit of quantification of vismodegib in plasma was 30 ng/mL.

The LC-MS/MS analysis was performed on a Xevo TQ-XS tandem quadrupole mass spectrometer (RRID:SCR_018510, Waters, Manchester, UK) attached with an Acquity UPLC I-Class system (Waters, Milford, MA, USA) and using an ESI-Spray interface as ion source. The parameters for the mass spectrometer were set to the following values: source temperature 150 °C, desolvation temperature 600 °C, desolvation gas flow rate 1000 l/h, cone gas flow rate 150 l/h, impactor voltage 3.1 kV, and detector gain of 1.

The liquid chromatography separation was performed on an Acquity UPLC BEH C18 column (130 Å, 1.7 µm, 2.1 × 50 mm) fitted with an Acquity UPLC BEH C18 VanGuard Pre-column (130Å, 1.7 µm, 2.1 × 5 mm) at a temperature of 45 °C. The mobile phases consisted of (A) 0.2% formic acid (LiChropur®, Sigma-Aldrich, Darmstadt, Germany) in water and (B) 0.2% formic acid in methanol. The experiment was run with gradient elution going from 5 B to 95% B over 3 min, followed by a 1-min washing step with 95% B and re-equilibration to initial conditions for 2 min. For the analysis, the mass spectrometer was operated in MRM mode in positive ion mode using the *m/z* 421→342 transition of vismodegib with a cone voltage of 2 V and a collision energy of 28 eV. Data acquisition and processing were performed in TargetLynx™ Application Manager (RRID:SCR_014271, Waters, Masslynx software, ver. 4.2, USA). Vismodegib concentrations were initially measured as ng vismodegib/mg of murine skin or ng vismodegib per milliliters of plasma. To convert these results to micromole per liter, we used the molecular weight of vismodegib (421.3 g/mol) and the density of rodent dorsal skin (1.076 g/cm^3^) [18]. Concentration results are reported as µmol vismodegib per liter in skin or plasma.

### Histology

For histology of AFL channels, we treated healthy skin with CO_2_-laser and isolated skin biopsies shortly after. For histology of microscopic BCCs, we isolated samples from non-treated tumor-induced skin. All skin biopsies were put into paraformaldehyde-soaked nylon filters (Leica Biosystems, product number 3801085, Maarn, the Netherlands) to reduce tissue stretching, placed in tissue cassettes, and submerged in stabilized and buffered 4% formaldehyde (VWR Chemicals, catalog number 9713.1000, Leuven, Belgium). Samples were then embedded in paraffin in a Shandon Excelsior ES (Thermo Fisher Scientific, Cheshire, UK) before being cut into 3-µm-thick sections with a RM2255 microtome (RRID:SCR_020229, Leica Biosystems, Nussloch, Germany). Tissue sections were stained with hematoxylin (Merck, mixed by Apoteket RegionH, Copenhagen, Denmark) and eosin (Acros, mixed by Apoteket, Copenhagen, Denmark), and microscopy and digitalization were performed on a Motic EasyScan Pro 6 (Motic, Barcelona, Spain) using a 20× objective.

### Statistics

**Fig. 2:**
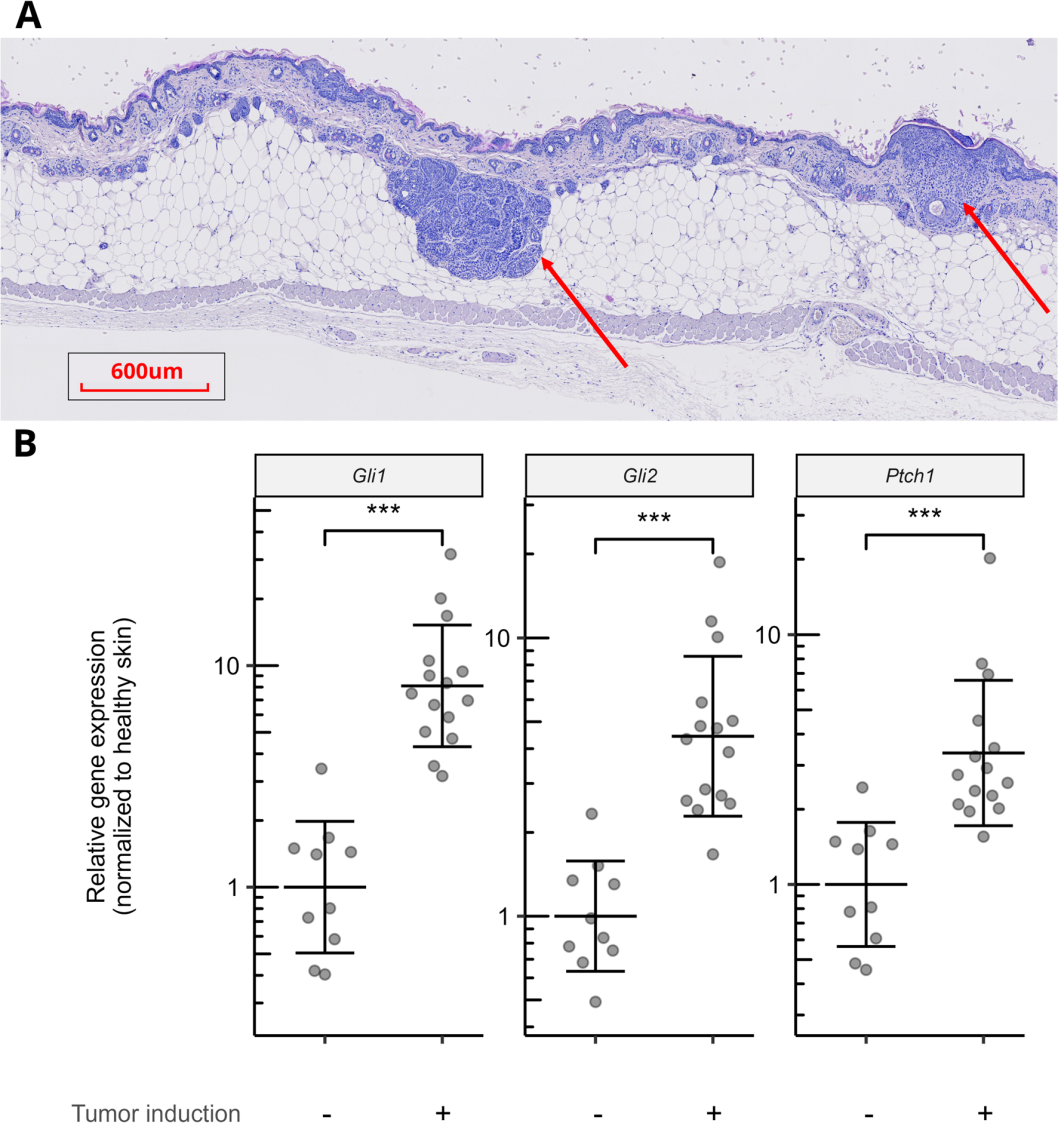
Microscopic BCCs in murine skin have upregulated hedgehog gene expression. (**A**) Histological slide showing tumor-induced murine skin with red arrows pointing at microscopic BCCs. (**B**) Expression levels of the hedgehog genes *Gli1*, *Gli2*, and *Ptch1* in healthy skin (*n* = 10) compared to tumor-induced skin (*n* = 15). Relative gene expression was normalized to the geometric mean of healthy skin samples. Each dot represents a skin test site. Geometric mean ± SD is represented by black lines. ***p < 0.001

Statistical analyses were performed with two-tailed Student *t*-tests (Figure 2B and Figure 5A) or linear regression (Figure 4 A–C and Table 1). For the linear regression, the treatments were coded as categorical variables and relative gene expression as outcome. Before linear regression, the relative gene expression was log10 transformed. Afterwards, estimates and confidence intervals were back-transformed and converted into percent reduction compared to non-treated tumor-induced skin. All outcomes were normally distributed, and the linear regression was checked for homogeneity of variance. All data visualization and statistical analyses were performed in R (RRID:SCR_001905, version 3.6.1, R Core Team, Vienna, Austria) and RStudio (RRID:SCR_000432, version 1.4.1717, PBC, Boston, MA, USA) using the following libraries: tidyverse [19] (RRID:SCR_019186) and patchwork [20]. We used a significance level of 0.05 for all comparisons.

**Table 1:** Percentage reduction of hedgehog gene expression by different treatments compared to tumor-induced skin

Treatment	Percentage gene expression reduction compared to tumor-induced skin (95% CI)		
	*Gli1*	*Gli2*	*Ptch1*
Tumor-induced skin	n/a	n/a	n/a
AFL alone	72.4 (37.8–87.8), *p* = 0.0027	55.2 (18.5–75.4), *p* = 0.0098	70.9 (45.1–84.5), *p* = 0.0003
Vismodegib alone	91.6 (78.8–96.6), *p* < 0.0001	83.3 (67.2–91.5), *p* < 0.0001	83.0 (65.3–91.7), *p* < 0.0001
Combination treatment	87.3 (69.2–94.8), *p* < 0.0001	75.1 (52.2–87.0), *p* = 0.0001	79.0 (58.2–89.5), *p* < 0.0001

*AFL* ablative fractional laser, *CI* confidence interval

## Results

### Tumor-induced murine skin with multiple microscopic BCCs

After tumor induction, multiple microscopic BCCs were present near the basement membrane of the murine skin (see histology in Figure 2A). The tumor-induced skin expressed mRNA levels of hedgehog genes *Gli1*, *Gli2*, and *Ptch1* that were 8.1, 4.4, and 3.4 times higher compared to healthy skin (all genes: *p* < 0.001, Figure 2B).

### AFL and vismodegib treatment reduced hedgehog gene expression

**Fig. 3:**
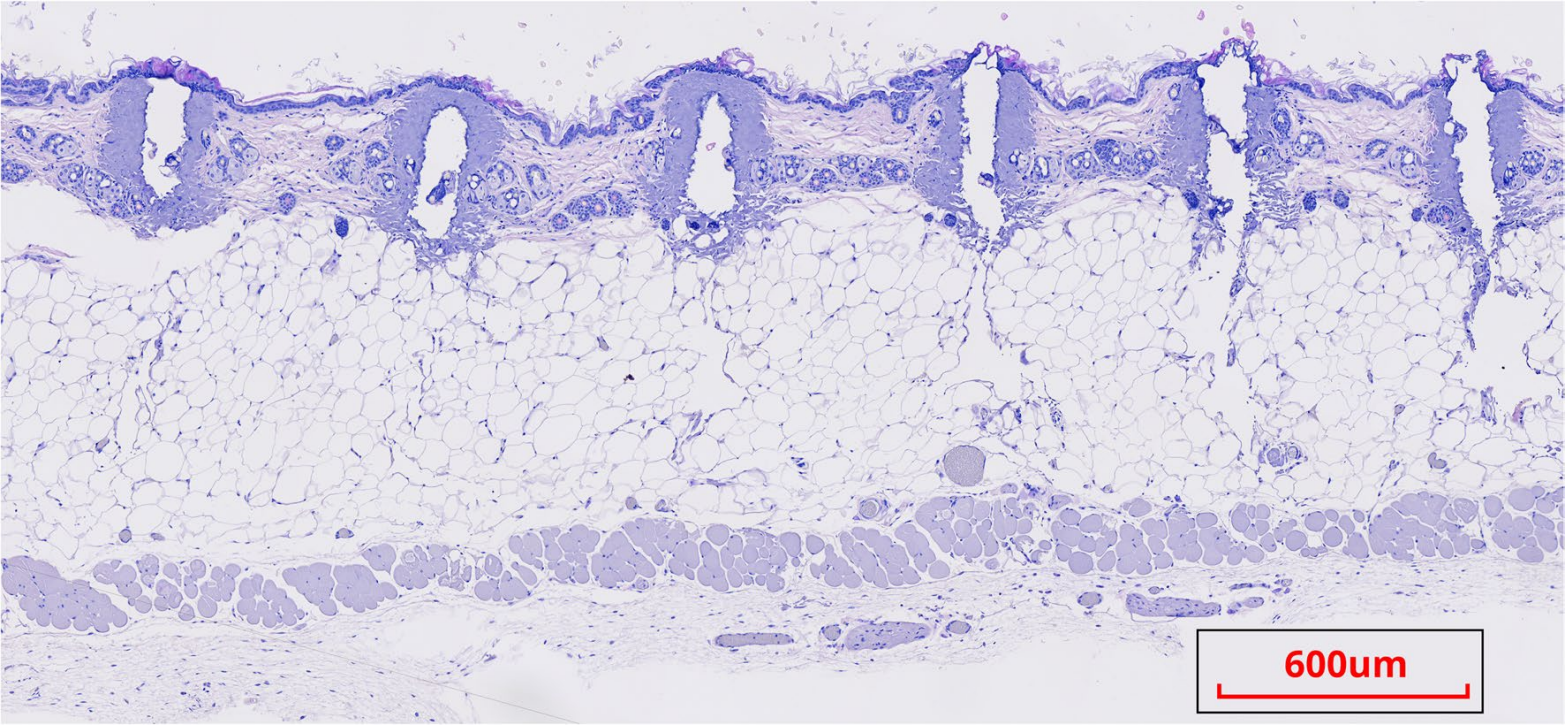
AFL channels in the skin. Histological slide of healthy skin treated with AFL (settings: 5% density 40 mJ/microbeam). AFL channels penetrated epidermis, dermis, and extended into hypodermis

**Fig. 4:**
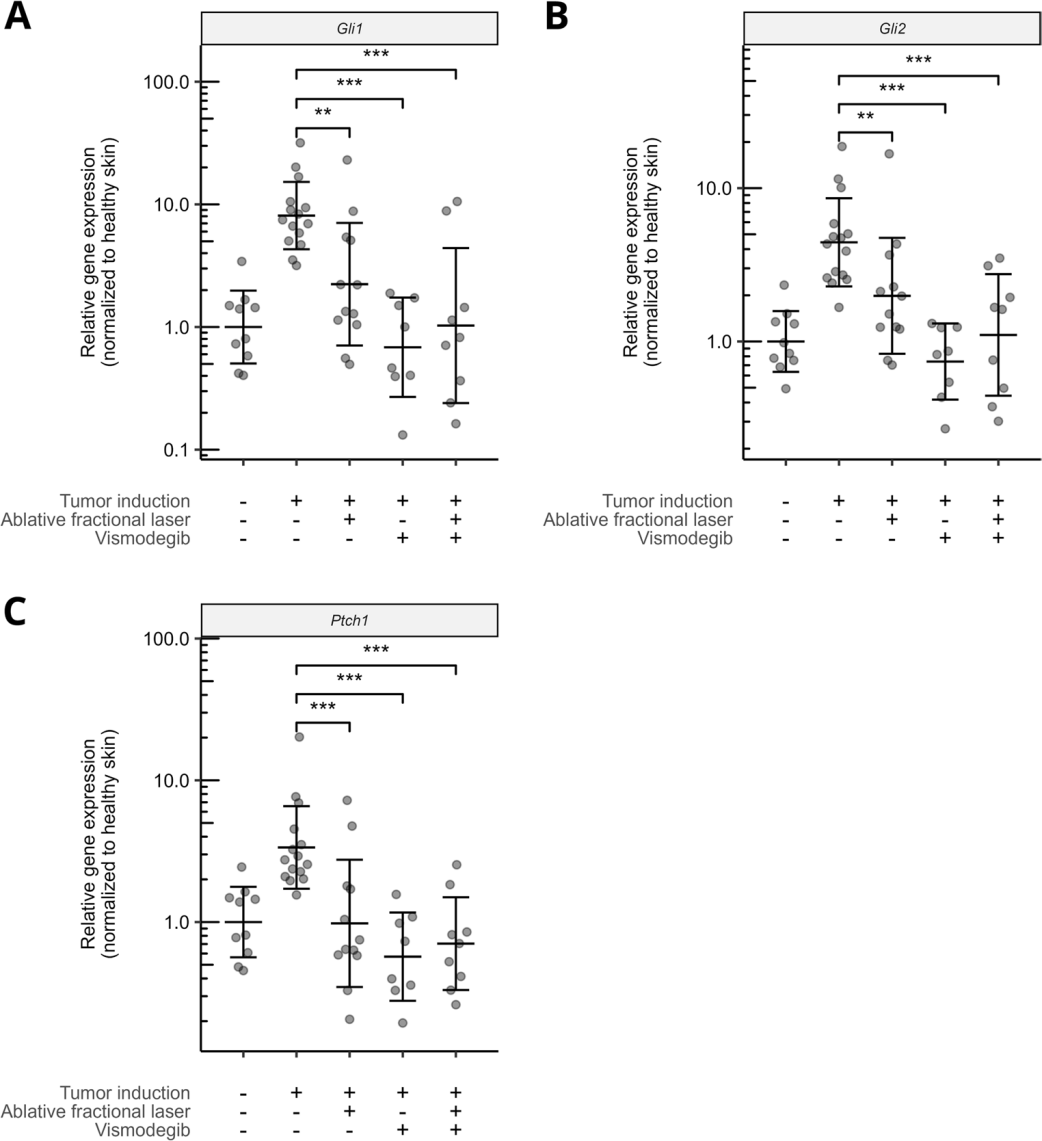
Skin with microscopic BCCs responded to AFL and vismodegib treatment. Expression levels of *Gli1* (**A**), *Gli2* (**B**), and *Ptch1* (**C**) in healthy skin (*n* = 10), tumor-induced skin (*n* = 15), AFL-treated tumor-induced skin (*n* = 12), vismodegib-treated tumor-induced skin (*n* = 8), and combination-treated tumor-induced skin (*n* = 9). Gene expression was normalized to the geometric mean of healthy skin samples. Each dot represents a skin test site. Geometric mean ± SD is represented by black lines. **p < 0.01, ***p < 0.001

In healthy skin, AFL treatment led to AFL channels that penetrated dermis and extended into hypodermis, passing the basement membrane where the microscopic tumors reside (Figure 3). In tumor-induced skin, a single treatment with AFL monotherapy resulted in significant mean reductions of mRNA expression of all three hedgehog genes (*Gli1*, 72.4%, *p* = 0.003; *Gli2*, 55.2%, *p* = 0.010; *Ptch1*, 70.9, *p* < 0.001; Figure 4A–C and Table 1). In comparison, the sequence of vismodegib treatments led to greater expression reduction of the three genes (*Gli1*, 91.6%; *Gli2*, 83.3%; *Ptch1*, 83.0), which in direct comparison with AFL monotherapy was significantly higher for two out of three hedgehog genes (*Gli1*, *p* = 0.017; *Gli2*, *p* = 0.007; *Ptch1*, *p* = 0.15). The combination of AFL and vismodegib resulted in expression reductions that equaled those achieved with vismodegib monotherapy (*Gli1*, 87.3%, *p* = 0.424; *Gli2*, 75.1%, *p* = 0.289; *Ptch1*, 79.0%, *p* = 0.593).

### Similar vismodegib concentrations in AFL-treated and non-exposed skin

**Fig. 5:**
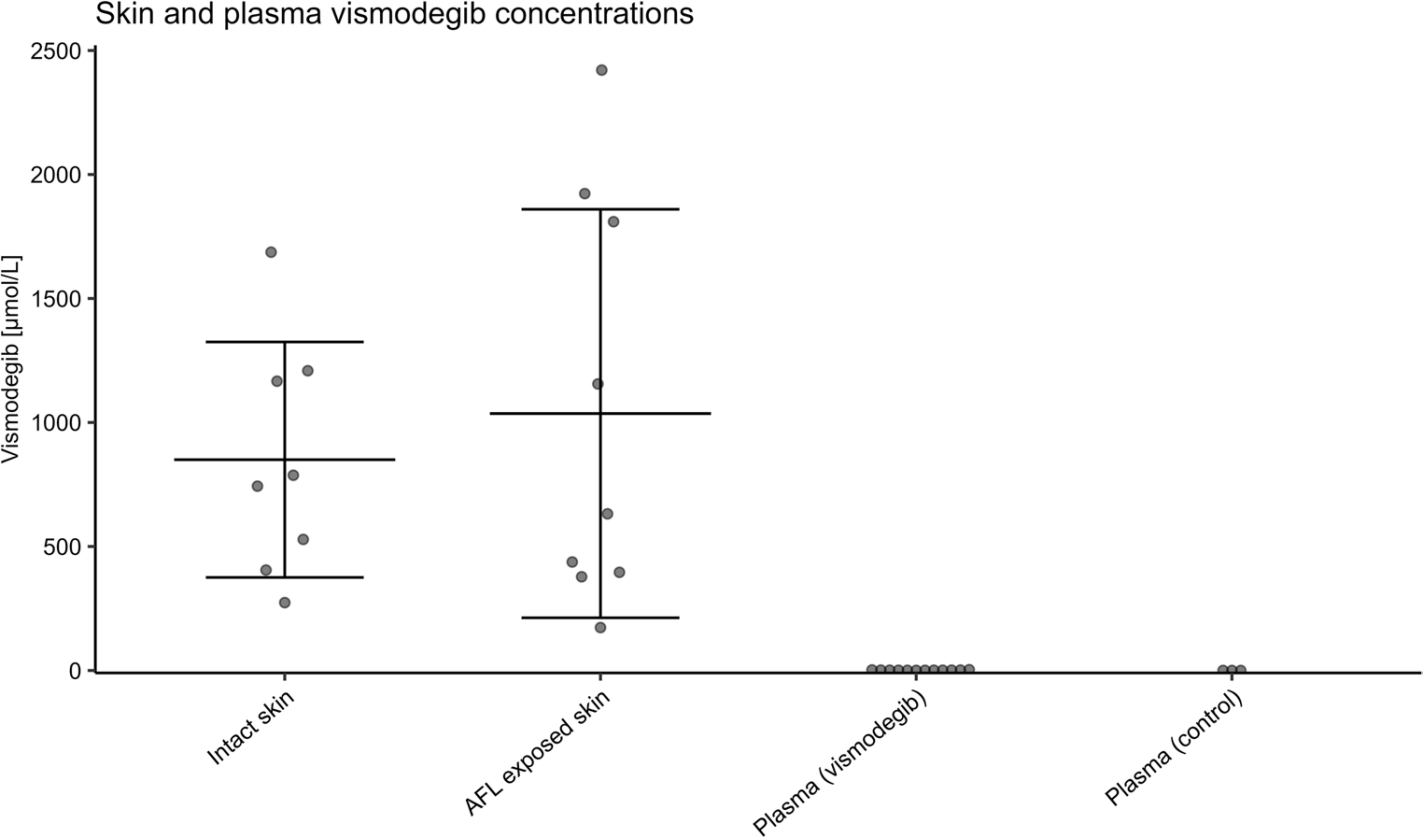
Vismodegib concentration in skin and plasma. In mice treated with vismodegib, uptake was quantified in intact skin (*n* = 8), AFL-exposed skin (*n* = 9), and in plasma samples (*n* = 12). Plasma from untreated mice was included as control (*n* = 3). Each dot represents a skin test site or an individual mouse. Mean ± SD is represented by black lines

Eight topical applications of vismodegib resulted in cutaneous vismodegib uptake (vismodegib monotherapy: 850 ± 475 µmol/L; Figure 5). Combination of AFL and vismodegib did not lead to further vismodegib uptake (combination treatment: 1036 ± 824 µmol/L, *p* = 0.573; Figure 5). Quantification of plasma from vismodegib-treated mice indicated minimal systemic uptake of vismodegib (1.14 ± 0.217 µmol/L; Figure 5).

## Discussion

In this study, we demonstrated that a single AFL treatment was able to reduce expression of hedgehog genes in microscopic murine BCCs. This AFL-induced reduction of hedgehog gene expression almost reached levels seen following eight topical vismodegib treatments. Because basal cell carcinomas depend on hedgehog signaling, AFL treatment could potentially be utilized to improve treatments of basal cell carcinomas, either as a monotherapy or in combination with other treatments.

We found that hedgehog gene expression was increased in murine skin with microscopic BCCs, and that both AFL- and vismodegib monotherapy as well as combination treatment can revert these changes. Comparison between AFL and vismodegib monotherapies revealed that vismodegib treatment led to the greatest reduction in hedgehog gene expression. This is consistent with the observed uptake of vismodegib, which in contrast to AFL, specifically targets hedgehog pathway activity. While the goal is to achieve a clinical response, e.g., by decreasing BCC volume, the threshold level of hedgehog gene expression required to induce such a response is uncertain. Other preclinical studies that used the same mouse model as we did have shown that 60–65% reduction of *Gli1* expression after 3 weeks was enough to reduce tumor size and, in some cases, lead to complete tumor regression [21]. In clinical trials, *GLI1* expression reductions of between 21 and 61.3% over 4 and 26 weeks mostly resulted in moderate decrease of tumor volume [22–26]. In comparison, AFL monotherapy reduced *Gli1* expression by 72.4%, which demonstrate the potentially substantial effect of AFL on skin and microscopic tumors. In accordance with our hypothesis, we show that AFL inhibits the hedgehog pathway; however, further studies are needed to determine whether this hedgehog inhibition can induce a clinical response in BCCs.

Histological slides from this study showed that the AFL channels penetrated the basement membrane and extended into hypodermis. The AFL channels reached the depth where the microscopic BCCs were located, but we have not elucidated by which mechanism AFL monotherapy reduces hedgehog gene expression. It is plausible that AFL monotherapy reduces hedgehog gene expression by ablating the microscopic tumors present in the skin, but since the density of the AFL treatment was 5%, ablation most likely does not fully account for the reduction of hedgehog gene expression reported in this study. Other studies have shown that AFL treatment impacts tissue homeostasis by modifying keratinocyte proliferation, immune cell recruitment, and fibroblast activation [9, 14, 27]. These changes also impact mRNA expression. For example, Spandau et al. demonstrated that fractionated laser resurfacing restored the IGF-1 mRNA expression level in geriatric skin, potentially by removing senescent fibroblasts [28]. Subsequent clinical studies on laser therapy of sun-damaged skin have shown that both non-ablative fractional lasers and AFL are able to protect against the development of keratinocyte carcinomas [12, 14]. The ability of AFL to reduce hedgehog gene expression reported in this study sheds new light on AFL’s ability to protect against skin cancer. An extensive review on cellular signaling pathways relevant for BCCs described crosstalk between the IGF-1 and the hedgehog signaling pathway [29]. Perhaps a single AFL monotherapy treatment can correct multiple age-related skin issues simultaneously leading to an efficient renewal of the skin that protects against all types of skin cancers.

The broad effects of AFL treatment might also be used to improve existing therapies by combining them with AFL treatment. In this study, we showed that substantial vismodegib uptake was achieved with eight topical applications of vismodegib monotherapy and that this uptake could not be further improved by the addition of AFL treatment. Compared to other studies that have tested topical application of vismodegib, the mean vismodegib concentrations achieved in this study (850 and 1036 µmol/L) are substantial. One study on in vivo pig skin achieved a cutaneous vismodegib level of 1409 µmol/L with AFL pre-treatment [16], and two studies on ex vivo human skin achieved cutaneous vismodegib levels of 15 and 20 µmol/L without pre-treatment [30, 31]. While AFL treatment was unable to improve vismodegib uptake in the present study, AFL treatment did enhance vismodegib delivery in the porcine skin [16], which is relevant for translation into human trials where cutaneous drug uptake is essential for treatment efficacy. We recently showed that combination of AFL and topical vismodegib was able to reduce hedgehog gene expression in human BCCs (manuscript accepted, Lasers in Surgery and Medicine, 2024). However, since AFL treatment can reduce hedgehog pathway activity on its own, it might perform well in combination with other drugs. For example, drugs like 5-fluoruacil and imiquimod, which have been approved for treatment of superficial BCCs [32], might improve their efficacy in combination with AFL treatment, because they are able to reduce hedgehog gene expression in addition to their other anti-tumor effects [33, 34].

The major limitations of this study are that we only investigate hedgehog gene expression at one time point and the substantial difference between murine and human BCCs. In future studies, investigations into the dynamics of hedgehog gene expression in response to AFL could help determine the best AFL treatment regimen. It is also important to verify whether human BCCs respond to AFL monotherapy, because the differences between human and murine BCCs, especially their size, could drastically change how they react to AFL treatment.

In conclusion, a single AFL treatment can lead to significant reduction of hedgehog gene expression, which opens a new avenue for potential BCC treatments. Further studies are needed to assess whether AFL is best utilized as a monotherapy or in combination with other treatments.

## Supplementary information

Below is the link to the electronic supplementary material.

Supplementary file1 (PDF 255 KB)
