# Supplementary material for: Ablative fractional laser treatment reduces hedgehog pathway gene expression in murine basal cell carcinomas
Source: Lasers Med Sci. 2024 Feb 3;39(1):55. doi: 10.1007/s10103-024-03997-1 (PMC10837214; doi:10.1007/s10103-024-03997-1)
Supplement: Supplementary file 1 — Supplementary file1 (PDF 255 KB) [file 10103_2024_3997_MOESM1_ESM.pdf]

Supplementary Information

Title: Ablative Fractional Laser Treatment Reduces Hedgehog Pathway Gene Expression in Murine Basal Cell Carcinomas

Authors:

Kristian Kåber Pedersen[a]* (0000-0002-9994-7848), Jonatan Riber Granborg[a] (0000-0002-5887-630X), Catharina Margrethe Lerche[a b] (0000-0003-3653-6424), Thomas Litman[c d] (0000-0002-6068-901X), Uffe Høgh Olesen[a] (0000-0002-4578-684X), Merete Hædersdal[a] (0000-0003-1250-2035)

[a]*Department of Dermatology, Copenhagen University Hospital – Bispebjerg, 2400 Copenhagen, Denmark*
[b]*Department of Pharmacy, University of Copenhagen, 2100 Copenhagen, Denmark*
[c]*Department of Immunology and Microbiology, University of Copenhagen, 2200 Copenhagen, Denmark*
[d]*Molecular Biomedicine, LEO Pharma A/S, 2750 Ballerup, Denmark*

**Corresponding author, email: kristian.kaaber.pedersen@regionh.dk*

Journal: Lasers in Medical Science (LIMS)

Supplementary Table 1 TaqMan qPCR probes used for the experiment

Gene	TaqMan Probe ID
Gli1	Mm00494654_m1
Gli2	Mm01293117_m1
Ptch1	Mm00436026_m1
Rplp0	Mm00725448_s1
Ppia	Mm02342430_g1
Rpl19	Mm02601633_g1

The probes were bought from Thermofischer (https://www.thermofisher.com/dk/en/home/life-science/pcr/real-time-pcr/real-time-pcr-assays/taqman-gene-expression.html)

Last accessed January 2, 2024
